# In vivo study of the effects of exogenous hydrogen sulfide on lung mitochondria in acute lung injury in rats

**DOI:** 10.1186/1471-2253-14-117

**Published:** 2014-12-15

**Authors:** Quansheng Du, Chao Wang, Nan Zhang, Guofeng Li, Meng Zhang, Liping Li, Qingzeng Zhang, Jianxin Zhang

**Affiliations:** Department of Pharmacology, Hebei Medical University, 361 Zhongshan Eastern Road, Shijiazhuang, 050017 China; Intensive Care Unit, Hebei General Hospital, 348 Heping Western Road, Shijiazhuang, 050051 China; Clinical research center, Hebei General Hospital, 348 Heping Western Road, Shijiazhuang, 050051 China; Department of Pharmacology, Children’s Hospital of Hebei Province, 133 Jianhua Southern Avenue, Shijiazhuang, 050031 China; Department of Pharmacology, Hebei Centers for Disease Control and Prevention, 97 Huaian Eastern Road, Shijiazhuang, 050021 China; Department of chest surgery, Third Hospital of Hebei Medical University, 139 Ziqiang Road, Shijiazhuang, 050051 China

**Keywords:** Acute lung injury, Mitochondria, Lipopolysaccharide, Exogenous hydrogen sulfide, Mitochondrial lipid peroxidation

## Abstract

**Background:**

Acute lung injury (ALI) is a serious disease with high incidence in ICU, and impaired mitochondria function plays a significant role in ALI. In this study, we examined the possible roles of exogenous hydrogen sulfide (H_2_S) in lung mitochondria regulation in ALI rats.

**Methods:**

The rat ALI model was induced by an intra-tongue vein Lipopolysaccharide (LPS) injection. We used sodium hydrosulphide (NaHS) as the H_2_S donor. We randomly divided 40 Sprague–Dawley rats into five groups: control, LPS injury, LPS + low-dose NaHS (0.78 mg•kg^-1^), LPS + middle-dose NaHS (1.56 mg•kg^-1^), and LPS + high-dose NaHS (3.12 mg•kg^-1^). Rats were killed 3 h after NaHS administration. We calculated a semi-quantitative histological index of lung injury assessments and measured the lung wet-to-dry weight ratio. We further analyzed serum for interleukin-1β levels using enzyme-linked immunosorbent assays. We observed lung mitochondria ultrastructures with an electron microscope. We examined oxidative stress markers in lung mitochondria and the mitochondrial swelling and activity. We analyzed lung mitochondria and cytosol Cyt-c protein expression using Western blotting.

**Results:**

Compared to the control group, the quantitative assessment score index, wet-to-dry weight ratios, and interleukin-1β content in the LPS injury group were significantly increased and the mitochondrial ultrastructure damaged. Furthermore, mitochondrial activity, adenosine triphosphatease, superoxide dismutase, glutathione peroxidase, and mitochondrial Cyt-c protein expression were significantly decreased, and malondialdehyde content, mitochondrial swelling, and cytosol Cyt-c protein expression were significantly increased in the LPS injury group compared to the control group. These effects were lessened by NaHS.

**Conclusion:**

Exogenous H_2_S provided a protective effect against ALI by decreasing the mitochondrial lipid peroxidation level and protecting the cell structure in the LPS-induced rat models. Its regulatory effect on lung mitochondria is positively correlated with the dosage.

## Background

Acute lung injury (ALI) and its most severe manifestation, acute respiratory distress syndrome (ARDS), is a clinical syndrome characterized by acute hypoxemic respiratory failure, bilateral pulmonary infiltrates on frontal chest radiograph consistent with edema, and normal cardiac filling pressures [1]. The resulting lung damage can evoke lung failure and multiple organ dysfunctions associated with increased mortality [2]. Sepsis (the presence of pus-forming bacteria or their toxins in the blood or tissues) is one of the most important ALI/ARDS causes [3]. Lipopolysaccharide (LPS), a major gram-negative bacillary endotoxin component, plays an important role in initiating inflammatory response and causing systemic inflammatory response syndrome (SIRS) and sepsis. The lung is one of the target organs primarily injured by endotoxin infection and sepsis. ALI induced by LPS is an acute pulmonary inflammation response in the lung, in which the accumulation and activation of polymophonuclear neutrophil (PMN) and oxygen free radical release are the key links [4]. LPS injection *in vivo* is a classic method to manufacture a sepsis-induced animal ALI model [5]. Inflammatory cell activation and increased oxidative stress are implicated in this pathogenesis [6]. Malondialdehyde [MDA], an end-product of membrane lipid peroxidation, adenosine triphosphatease [ATPase], anti-oxidants superoxide dismutase [SOD], and glutathione peroxidase [GPx] are currently considered the basic oxidative stress markers.

LPS damages the mitochondrial structure, and the ATP enzyme and oxidative phosphorylation coupling process leads to energy metabolism disorders. LPS-induced-ALI can cause abnormal mitochondrial structures and functions, and abnormalities tend to change other cell organelles and the whole cell, thereby increasing ALI’s degree [7]. Studies have shown that oxidant-induced death and dysfunction of pulmonary vascular cells play important roles in ALI’s evolution, and oxygen radicals that damage DNA in their mitochondria play an important role in its process [8]. A cell’s energy sub-units mitochondria are significant to biological energy metabolism. When subjected to outside influences, its dysfunction may be an important lung injury component. Mitochondrial dysfunction may be a main cause for ALI, so improving mitochondrial function may be important for treating ALI.

In recent years, hydrogen sulfide (H_2_S) has been seen as very important in a wide range of physiological and pathological functions [9–13]. Recently, some experiments confirmed that endogenous H_2_S played an important role in inflammatory disease pathogenesis and associated organ injury, such as acute pancreatitis [14], sepsis [15], ischemia/reperfusion injury [16], and lung injury (ventilator-induced lung injury [17], or oleic acid-induced ALI [18]), succeeded in exerting organ protective effects. Some experiments have shown that H_2_S may participate in ALI/ARDS pathogenesis. However, few experiments focus on exogenous H_2_S (inhalational or parenteral administration) [2, 18, 19], so H_2_S-related therapy for ALI/ARDS may be a potential therapeutic approach [20]. Also, injecting NaHS (exogenous H_2_S) in normal rats directly results in lung inflammation and inflammatory damage in a dose-dependent manner [21]. It is possible that H_2_S affects the ALI/ARDS pathogenesis through mitochondria. Thus, our study aims to find the possible roles of H_2_S in LPS-induced ALI in rats and its regulatory effects on the lung mitochondria with different doses.

## Methods

This study was approved by the Institutional Experimental Animal Care and Ethics Committee of Hebei Medical University (Shijiazhuang, China). All animal care and experimental protocols complied with the Animal Management Rules of the Ministry of Health of the People’s Republic of China (documentation number 19890503) and the Guide for the Care and Use of Laboratory Animals of Hebei Medical University (Shijiazhuang, China). The study was performed in the Animal Laboratory of our Department of Pharmacology.

### Animals

This study included 40 male Sprague–Dawley rats, weighing 250–280 g, which were obtained from the Experimental Animal Centre, Hebei Medical University (Shijiazhuang, China) and kept in our Laboratory Animal Husbandry Facility until the experiments. Rats were acclimated for 1 week before experiments, with unrestricted access to deionized water and standard rat chow with no other restrictions. Before and throughout the study, rats were kept at room temperature (22°C) at 30%–70% humidity on a 12 h day/night cycle with the lights turned on at 7:00 AM. No deaths occurred before intervention. All procedures were performed in accordance with the Guidelines of Animal Experiments from the Committee of Medical Ethics, National Health Department of China.

### Materials

The H_2_S donor NaHS was obtained from Sigma-Aldrich (St Louis, MO, USA). E.coli LPS (serotype 0127:B8) was obtained from the Sigma-Aldrich Company, Ltd. (Poole, Dorset, U.K.). SOD, MDA, GPx, and ATPase detection kits were obtained from the Nanjing Jiancheng Bioengineering Institute (Nanjing, Jiangsu, China). The primary rabbit multiclonal antibodies were obtained from Abcam Company, Ltd. (Cambridge, U.K.). All stock solutions were prepared in nonpyrogenic saline (0.9% NaCl) from Shijiazhuang No.4 Pharmaceutical Company, Ltd. (Shijiazhuang, Hebei, China).

### Experimental protocol

Rats were randomly divided into five groups (n = 8 in each): control, LPS injury, LPS + low-dose NaHS (0.78 mg•kg^-1^), LPS + middle-dose NaHS (1.56 mg•kg^-1^), and LPS + high-dose NaHS (3.12 mg•kg^-1^). Rats were anaesthetized with an injection of 100 g/L chloral hydrate (3 ml•kg^-1^). Anesthesia depth was evaluated every 2–3 min throughout the study. No supplemental oxygen, fluids, or mechanical ventilation were involved during the anesthesia period. The same dose was administered to the different groups. After adequate anesthesia, rats were fixed in the dorsal position on a surgical table.

1. Control group: Rats were treated with an equal volume of saline without causing endotoxemia.
2. LPS injury group: Rats were treated with E.coli LPS (5 mg•kg^-1^ via sublingual vein injections, serotype 0127:B8) lasting over 10 min and also treated with an equal volume of saline via intraperitoneal injections 3 h after LPS induction.
3. LPS + low-dose NaHS group: Rats were administered NaHS (0.78 mg•kg^-1^) via intraperitoneal injection 3 h after LPS induction.
4. LPS + middle-dose NaHS group: Rats were administered NaHS (1.56 mg•kg^-1^) via intraperitoneal injection 3 h after LPS induction.
5. LPS + high-dose NaHS group: Rats were administered NaHS (3.12 mg•kg^-1^) via intraperitoneal injection 3 h after LPS induction.

All rats were killed 3 h after NaHS or saline administration.

### Lung histology and IQA

The middle lobe of the right lung was fixed in 10% (wt/vol) formalin and routinely processed in paraffin sections for hematoxylin and eosin staining. Ten fields from each slice were visualized by microscopy (×200). The average values were taken as a modified semi-quantitative histological index of quantitative assessment (IQA) of lung injury using the following criteria: 0, no alveolitis; +1, thickening of the alveolar septum by a mononuclear cell infiltrate, with involvement limited to focal, pleural-based lesions occupying less than 25% of the lung and with good alveolar architecture preservation; +2, more extensive fibrosis involving 26%-50% of the lung and fibrotic regions, mostly extending inward from the pleura and still focal; +3, widespread fibrosis involving 51%-70% of the lung; and +4, widespread fibrosis involving more than 70% of the lung [22, 23].

### Determination of wet-to-dry lung weight ratio

The lung wet-to-dry (W/D) weight ratio was used as an index of lung water accumulation. To measure the total amount of lung water, we measured the right lung upper lobe weight immediately after its excision (wet weight). The lung tissue was then dried in an oven at 60°C for 48 h and re-weighed as dry weight. The W/D weight ratio was calculated by dividing the wet by the dry weight, as described previously.

### Cytokine measurements

Blood was drawn from the left carotid artery, and serum samples were frozen and stored at -80°C. Serum samples were tested using ELISA kits for interleukin-1β (IL-1β) (R&D Systems GmbH, Wiesbaden, Germany) according to the manufacturers’ instructions.

### Observation of ultrastructural changes to mitochondria in lung cells

After rats were killed, the lung tissue (1 mm^3^) was resected and fixed in 4% glutaraldehyde in 0.1 mol•L^-1^ phosphate buffer (pH 7.4) at 4°C. Tissues were washed three times in dimethyl arsenate buffer and then post-fixed with 1% osmium tetroxide for 1 h, followed by another three washes in dimethyl arsenate buffer and dehydration by passage through graded ethylene alcohol concentrations. After sequential propylene oxide treatments, ultrathin sections were cut using a Leica (Wetzlar, Germany) UCT Ultra Microtome, stained with 1% uranyl acetate and lead citrate, and observed under a transmission electron microscope.

### Isolated mitochondria preparation

Mitochondria were isolated as previously described. Briefly, after rats were killed, the lung tissues were removed rapidly into ice-cold isolation medium (0.025 mol•L^-1^ sucrose, 0.075 mol•L^-1^ mannitol, 0.001 mol•L^-1^ EDTA, and pH 7.4). The tissues were finely homogenized using a homogenizer in a glass pestle. The homogenate was centrifuged at 600 g for 7 min at 2°C. The supernatant was collected and then centrifuged again at 1 600 g for 5 min at 2°C. The crude mitochondrial pellet was resuspended in a final volume of 5 mL of 3% Ficoll medium (0.12 mol•L^-1^ mannitol, 0.03 mol•L^-1^ sucrose, 0.025 mol•L^-1^ K^+^-EDTA, pH 7.4). This suspension was carefully layered onto 10 mL of 6% Ficoll medium (0.24 mol•L^-1^ mannitol, 0.06 mol•L^-1^ sucrose, 0.05 mol•L^-1^ K^+^-EDTA, pH 7.4) and centrifuged at 12 500 g for 10 min at 2°C. The supernatant was decanted and the slight fluffy layer removed from the pellet. The mitochondrial pellet was resuspended in isolation medium and centrifuged again at 12 500 g for 10 min at 2°C. The prepared mitochondria were diluted in isolation medium prior to use.

### Determining lung mitochondrial MDA content, SOD, and GPx and ATPase activity

SOD levels, GPx activity, ATPase activity, and MDA content in the lung mitochondria were measured with an enzyme-linked immunosorbent assay (ELISA) and commercially available kits (Nanjing Jiancheng Bioengineering, Nanjing, Jiangsu, China) according to the manufacturer’s instructions. The kit was maintained at room temperature (20–25°C) prior to testing and the washing buffer prepared 15 min before use. The 100 μl of SOD standards and 100 μl of samples were added to the corresponding plate wells and shaken gently for 30 s before sealing. After 1 h of incubation at 37°C, the liquid was removed, and the plates were washed with washing buffer (350 μl/well) and soaked for a few minutes. Plates were blotted dry by tapping upside down on filter paper. We repeated the washing five times. The 100 μl of biotin was added to each plate well and left for 1 h at 37°C. After five additional washing steps, 100 μl of horseradish peroxides (HRP) was added to the wells and left for 30 min at 37°C. The plates were again washed five times, 100 μl of tetramethylbenzidine (TMB) substrate was added to each well, and plates were shaken gently for 10 s. The mixture was incubated in the dark for (15 ± 10) min at 37°C. Optical density (OD) at 450 nm was measured by ELISA reader after adding 100 μl of stop solution to each well and shaking gently for 30 s. The standard curve of OD value versus concentration was plotted. The sample data were plotted on the standard curve and the sample SOD concentration obtained. Other samples were obtained in a similar way.

### Determining lung mitochondria swelling

Freshly prepared mitochondria without repeated freeze-thaw were kept at 4°C prior to the reaction. The mitochondria were removed rapidly into the medium (0.025 mol•L^-1^ sucrose, 0.0005 mol•L^-1^ KH_2_PO_4_, 0.001 mol•L^-1^ Sodium succinate, and pH 7.2). Mitochondrial protein content was adjusted to 0.5 mg•ml^-1^. Mitochondrial swelling was determined in all groups by measuring the change in the absorbance of the lung mitochondrial suspension at 540 nm (Optical density 540,OD540) on a spectrophotometer at 722. The reaction conditions were set at 25°C.

### Determining of the lung mitochondria activity

Freshly prepared mitochondria suspension (100 μL) without repeated freeze-thaw was removed rapidly into the microtiter plate’s microporous. We added MTT (5 g•L^-1,^ 40 μL) at 30°C and incubated it for 30 min, then added isopropanol (100 μL) for 20 min. Colorimetric analysis was set at 570 nm. OD570 value size indicated mitochondrial activity.

### Determining lung mitochondrial and cytosol Cyt-c protein expression

After rats were killed, lung tissues were removed rapidly into ice-cold isolation medium (0.025 mol/L sucrose, 0.075 mol/L mannitol, 0.001 mol/L EDTA, and pH 7.4). The tissues were finely homogenized using a homogenizer in a glass pestle. The homogenate was centrifuged at 600 g for 7 min at 2°C. The supernatant was collected and then centrifuged again at 1 600 g for 5 min at 2°C. The crude mitochondrial pellet was resuspended in a final volume of 5 mL of 3% Ficoll medium (0.12 mol/L mannitol, 0.03 mol/L sucrose, 0.025 mol/L K^+^-EDTA, pH 7.4). This suspension was carefully layered onto 10 mL of 6% Ficoll medium (0.24 mol/L mannitol, 0.06 mol/L sucrose, 0.05 mol/L K^+^-EDTA, pH 7.4) and centrifuged at 12 500 g for 10 min at 2°C. The supernatant was decanted and the slight fluffy layer removed from the pellet. The mitochondrial pellet was resuspended in isolation medium and centrifuged again at 12 500 g for 10 min at 2°C. The mitochondria and cytosol supernatant were used for cytochrome c (Cyt-c) analysis using Western blotting assay.

Protein extractions in each group were subjected to 10% sodium dodecyl sulfate-polyacrylamide gel electrophoresis (SDS-PAGE) and transferred onto a polyvinylidene difluoride (PVDF) membrane. The membranes were treated with blocking solution (5% skim milk in TBST) and incubated overnight at 4°C with the primary rabbit multiclonal antibodies, respectively (Cyt-c, 1:1000; abcam, U.K.). Immunoblots were developed with horseradish peroxidase-conjugated secondary antibodies, visualized using an enhanced chemiluminescence reagent (Millipore, Billerica, MA, USA), and quantified by densitometry using a ChemiDoc XRS (Bio-Rad, Berkeley, California, USA). The band density was normalized to β-actin. The proteins’ increase or decrease percentage was estimated by comparison to vehicle control (100%).

### Statistical analysis

Based on the assumed differences and variability in the data marking a biologic effect of treatment in each group, numbers of animals per group were estimated before the study. All data are presented as the mean ± SD. Differences between groups were assessed by one-way ANOVA followed by Tukey’s post hoc multiple comparison test using SPSS, version 13.0 (SPSS, Armonk, NY, USA). IQA scores were achieved using the rank-sum test. Two-sided P values of less than 0.05 were considered statistically significant.

## Results

### Histological lung tissue changes in each group

**Figure 1:**
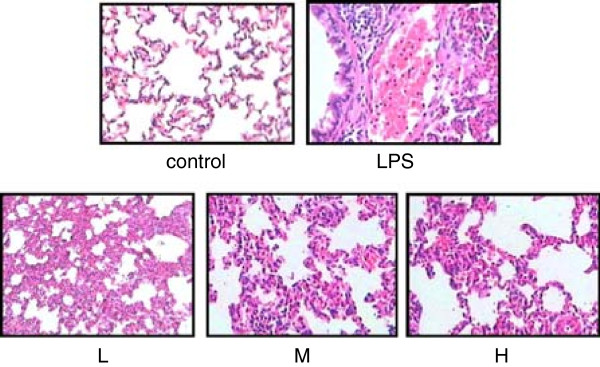
**Histologic changes of lung tissue in each group.** Microphotographs of morphological changes of lung tissues (original magnification × 200). Hematoxylin and eosin staining:control: control group. LPS: LPS injury group. L: LPS + low-dose NaHS group. M: LPS + middle-dose NaHS group. H: LPS + high-dose NaHS group. In comparison, the LPS injury group showed diffuse edema in alveolar spaces and interstitium of the lung, hemorrhage, severe inflammatory cell infiltration and serous exudation in the alveolar space, and thickened interbular septa. These changes were lightly mitigated in the LPS + low-dose NaHS group, and were significantly mitigated in LPS + middle-dose NaHS group and high-dose NaHS group.

We found that the control group morphology was normal with light microscopy, and there was no alveolar edema fluid in the alveolar space. The alveolar wall was intact with no evidence of inflammatory cell infiltration or hemorrhage. Conversely, the LPS injury group showed diffuse edema in alveolar spaces, lung interstitium, hemorrhage, severe inflammatory cell infiltration, serous exudation in the alveolar space, and a thickened interbular septa like “hyaline membrane”. These changes were lightly mitigated in the LPS + low-dose NaHS group, and significantly mitigated in LPS + middle-dose and high-dose NaHS groups (Figure 1).

### IQA scores in each group

Significantly higher IQA scores were observed in the LPS group (3.12 ± 0.02 vs. 0.05 ± 0.08, *P* < 0.01) compared to the control group, but IQA scores were slightly lower in the LPS + low-dose NaHS group (2.52 ± 0.12 vs. 3.12 ± 0.02, *P* < 0.01) and significantly lower in the LPS + middle-dose (2.47 ± 0.20 vs. 3.12 ± 0.02, *P* < 0.01) and high-dose NaHS groups (2.32 ± 0.15 vs. 3.12 ± 0.02, *P* < 0.01) compared to the LPS group.

### W/D lung weight ratios

The W/D lung weight ratios were significantly increased in the LPS group (5.52 ± 0.20 vs. 4.73 ± 0.32, *P* < 0.01) compared to the control group. Compared to the LPS group, the W/D lung weight ratios were respectively decreased in LPS + low-dose (5.20 ± 0.27 vs. 5.52 ± 0.20, *P* < 0.05), middle-dose (4.69 ± 0.41 vs. 5.52 ± 0.20, *P* < 0.01), and high-dose (4.84 ± 0.22 vs. 5.52 ± 0.20, *P* < 0.01) NaHS groups.

### IL-1β content in serum

Compared to the control group, the IL-1β serum content (15.03 ± 0.82 pg/ml vs. 10.58 ± 1.06 pg/ml, *P* < 0.01) was significantly increased in the LPS group. Compared to the LPS group, the IL-1β serum content was significantly deceased in the LPS + low-dose (13.96 ± 0.72 pg/ml vs. 15.03 ± 0.82 pg/ml*, P* < 0.05), middle-dose (12.46 ± 1.33 pg/ml vs. 15.03 ± 0.82 pg/ml*, P* < 0.01), and high-dose (12.23 ± 0.89 pg/ml vs. 15.03 ± 0.82 pg/ml*, P* < 0.01) NaHS groups.

### Effects of H_2_S on mitochondrial ultrastructure in lung cells

**Figure 2:**
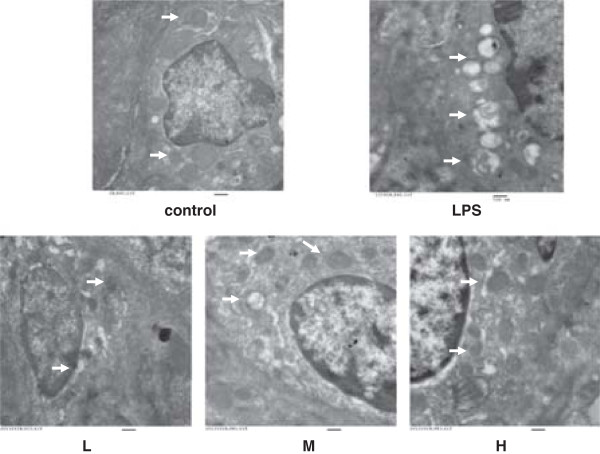
**Lung Ultrastructure.** Effect of H_2_S on mitochondrial ultrastructure in lung cells, as determined by transmission electron microscopy analysis, in LPS-Induced ALI rat model (original magnification × 25000). There were significant differences in the ultrastructure of mitochondria in lung cells in the control group (control) and LPS injury group (LPS). The mitochondria in lung cells from the LPS group were swollen with disrupted or disintegrated cristae and the osmiophilic lamellar bodies were fusion or disappeared. This mitochondrial damage was lightly mitigated in the LPS + low-dose NaHS group (L), and were significantly mitigated in LPS + middle-dose NaHS group (M) and high-dose NaHS group (H). Arrows indicate mitochondria in lung cells. Bar, 500 nm.

There were significant differences between the mitochondrial ultrastructure in the control and LPS groups’ lung cells. The mitochondria in lung cells from the LPS injury group were swollen with disrupted or disintegrated cristae, and the osmiophilic lamellar bodies had fused or disappeared. This mitochondrial damage was slightly mitigated in the LPS + low-dose NaHS group and significantly mitigated in the LPS + middle-dose and high-dose NaHS groups (Figure 2).

### Effects of H_2_S on MDA content and mitochondrial ATPase, SOD, and GSH-P_X_ activity

**Figure 3:**
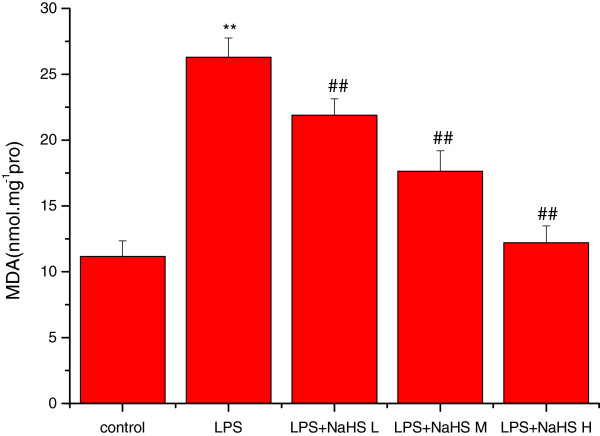
**Effect of H**_**2**_ **S on the content of MDA in lung mitochondria in rats.** Compared with control group, the content of MDA was significantly increased (*P* < 0.01) in lung mitochondria in LPS injury group. Compared with LPS injury group, the content of MDA was significantly decreased in LPS + low, middle and high dose NaHS groups (*P* < 0.01). **P < 0.01 *vs* control; ##*P* < 0.01 *vs* LPS. control: control group. LPS: LPS injury group.LPS+ NaHS L: LPS + low-dose NaHS group.LPS+ NaHS M: LPS + middle-dose NaHS group.LPS+ NaHS H: LPS + high-dose NaHS group.

**Figure 4:**
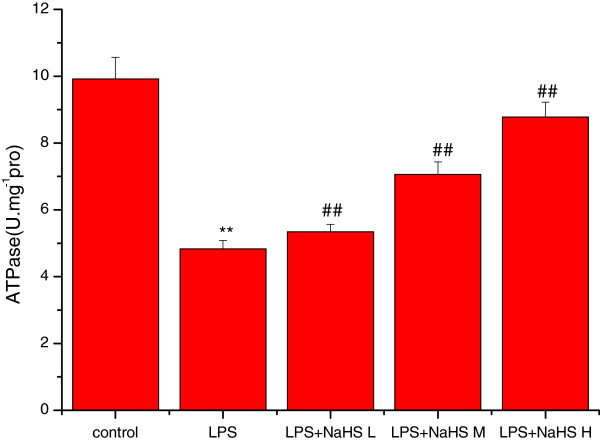
**Effect of H**_**2**_ **S on the activity of ATPase in lung mitochondria in rats.** Compared with control group, the activities of ATPase were significantly decreased (*P* < 0.01) in lung mitochondria in LPS injury group. Compared with LPS injury group, the activities of ATPase were significantly increased in LPS + low, middle and high dose NaHS groups (*P* < 0.01). **P < 0.01 *vs* control; ##*P* < 0.01 *vs* LPS.control: control group. LPS: LPS injury group.LPS+ NaHS L: LPS + low-dose NaHS group.LPS+ NaHS M: LPS + middle-dose NaHS group.LPS+ NaHS H: LPS + high-dose NaHS group.

**Figure 5:**
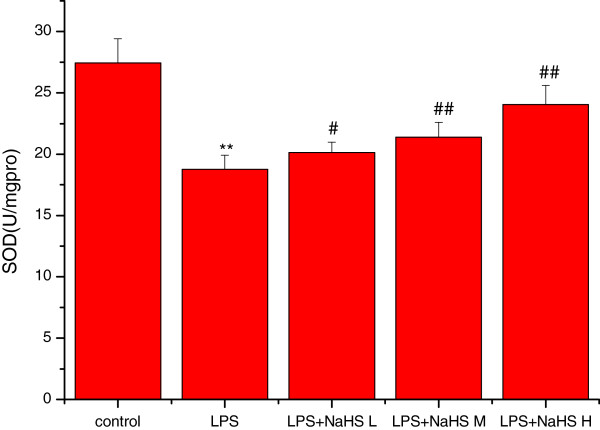
**Effect of H**_**2**_ **S on the activity of SOD in lung mitochondria in rats.** Compared with control group, the activities of SOD were significantly decreased (*P* < 0.01) in lung mitochondria in LPS injury group. Compared with LPS injury group, the activities of SOD were significantly increased in LPS + low, middle and high dose NaHS groups (*P* < 0.05 or *P* < 0.01). **P < 0.01 *vs* control; #*P* < 0.05, ##*P* < 0.01 *vs* LPS.control: control group. LPS: LPS injury group.LPS+ NaHS L: LPS + low-dose NaHS group.LPS+ NaHS M: LPS + middle-dose NaHS group.LPS+ NaHS H: LPS + high-dose NaHS group.

**Figure 6:**
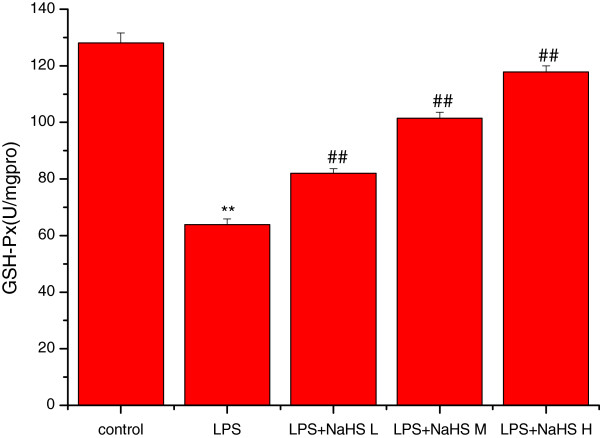
**Effect of H**_**2**_ **S on the activity of GSH-P**_**X**_ **in lung mitochondria in rats.** Compared with control group, the activities of GSH-P_X_ were significantly decreased (*P* < 0.01) in lung mitochondria in LPS injury group. Compared with LPS injury group, the activities of GSH-P_X_ were significantly increased in LPS + low, middle and high dose NaHS groups (*P* < 0.01). **P < 0.01 *vs* control; ##*P* < 0.01 *vs* LPS.control: control group. LPS: LPS injury group.LPS+ NaHS L: LPS + low-dose NaHS group.LPS+ NaHS M: LPS + middle-dose NaHS group.LPS+ NaHS H: LPS + high-dose NaHS group.

Compared to the control group, the MDA content was significantly increased (*P* < 0.01) and SOD, GSH-PX, and ATPase activities significantly decreased (*P* < 0.01) in lung mitochondria in the LPS group. Compared to the LPS group, the MDA content was significantly decreased and SOD, GSH-PX, and ATPase activities significantly increased in the three LPS + NaHS groups (*P* < 0.05 or *P* < 0.01) (Figures 3, 4, 5 and 6).

### Effects of H_2_S on lung mitochondria swelling and activity

**Figure 7:**
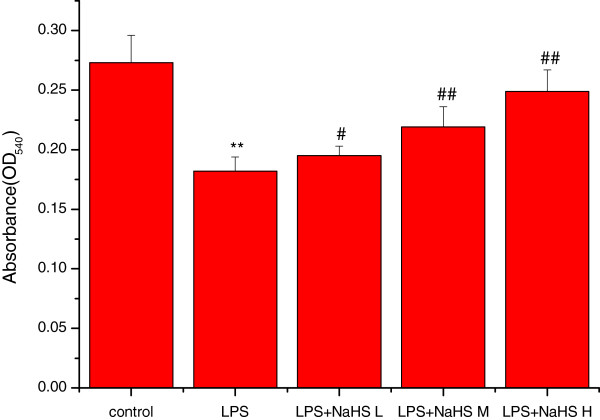
**Effect of H**_**2**_ **S on the swelling of lung mitochondria in rats.** The swelling extent of the mitochondria was significantly increased (the OD540 value was significantly decreased) in the LPS injury group compared with the control group (*P* < 0.01). In LPS + low-dose NaHS group, LPS + middle-dose NaHS group and LPS + high-dose NaHS group, the swelling of the mitochondria was markedly decreased (the OD540 value was significantly increased) with the LPS injury group (*P* < 0.05 or *P* < 0.01). **P < 0.01 *vs* control; #*P* < 0.05, ##*P* < 0.01 *vs* LPS.control: control group. LPS: LPS injury group.LPS+ NaHS L: LPS + low-dose NaHS group.LPS+ NaHS M: LPS + middle-dose NaHS group.LPS+ NaHS H: LPS + high-dose NaHS group.

**Figure 8:**
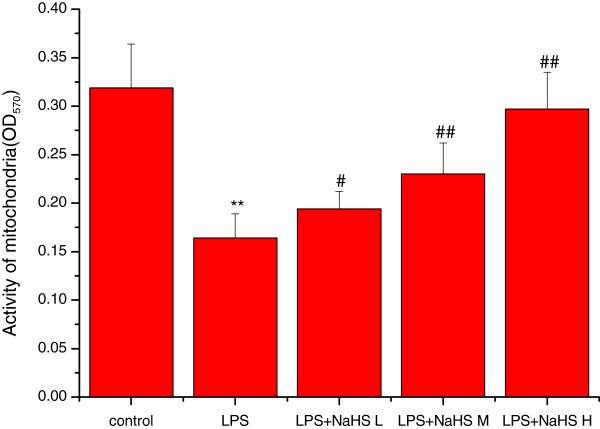
**Effect of H**_**2**_ **S on the activity of lung mitochondia in rats.** The activity of the mitochondria was significantly decreased in the LPS injury group compared with the control group (*P* < 0.01). In LPS + low-dose NaHS group,LPS + middle-dose NaHS group and LPS + high-dose NaHS group, the activity of the mitochondria was markedly increased compared with the LPS injury group (*P* < 0.05 or *P* < 0.01). **P < 0.01 *vs* control; #*P* < 0.05, ##*P* < 0.01 *vs* LPS.control: control group. LPS: LPS injury group.LPS+ NaHS L: LPS + low-dose NaHS group.LPS+ NaHS M: LPS + middle-dose NaHS group.LPS+ NaHS H: LPS + high-dose NaHS group.

Mitochondrial permeability transition (MPT) is an important index to indicate the integrity and function of mitochondria.The principle of measurement for MPT is to observe the change of absorbance at 540 nm (OD540), due to the swelling of mitochondria by the disturbance of permeability transition of mitochondrial membrane when MPT increase. Decreased absorbance (the OD540 value) indicates mitochondrial swelling [24, 25]. Mitochondrial activity was assessed by the MTT colorimetric assay using OD570. The swelling of the mitochondria was significantly increased (the OD540 value was significantly decreased) and the activity of the mitochondria was significantly decreased in the LPS compared with the control group (P < 0.01). In the three LPS + NaHS groups, the swelling of the mitochondria was markedly decreased (the OD540 value was significantly increased) and the activity of the mitochondria was markedly increased compared with the LPS group (P < 0.05 or P < 0.01) (Figures 7 and 8).

### Effects of H_2_S on lung mitochondrial and cytosol Cyt-c protein expression

**Figure 9:**
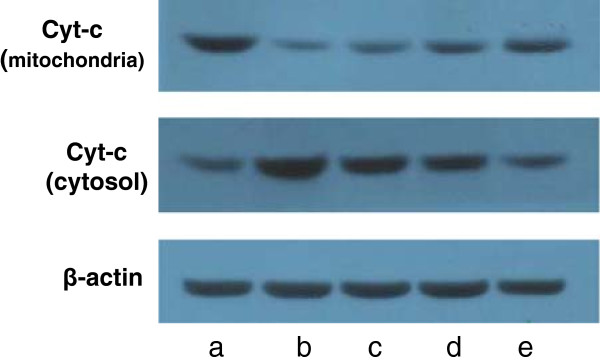
**The expression of lung mitochondrial Cyt-c and cytosol Cyt-c were detected by Western blotting method.** The Cyt-c protein expression of the lung mitochondria was significantly decreased in the LPS injury group compared with the control group (*P* < 0.01). In the LPS + low-dose, middle-dose and high-dose NaHS group, Cyt-c protein expression of the lung mitochondria was markedly increased compared with the LPS injury group (*P* < 0.05 or *P* < 0.01). The Cyt-c protein expression of cytosol was significantly increased in the LPS compared with the control group (*P* < 0.01). In the LPS + low-dose, middle-dose and high-dose NaHS group, Cyt-c protein expression of cytosol was markedly decreased compared with the LPS injury group (*P* < 0.05 or *P* < 0.01). **a**: control group. **b**: LPS injury group. **c**: LPS+ low-dose NaHS group. **d**: LPS + middle-dose NaHS group. **e**: LPS + high-dose NaHS group.

The band intensity showed the Cyt-c protein expression. The Cyt-c protein expression in the lung mitochondria was significantly decreased in the LPS injury group compared to the control group (*P* < 0.01). In the three LPS + NaHS groups, Cyt-c protein expression in the lung mitochondria was markedly increased compared to the LPS injury group (*P* < 0.01). The cytosol Cyt-c protein expression was significantly increased in the LPS group compared to the control group (*P* < 0.01). In the three LPS + NaHS groups, cytosol Cyt-c protein expression was markedly decreased compared to the LPS injury group (*P* < 0.01) (Table 1, Figure 9).

**Table 1:** **Effects of NaHS on lung mitochondrial and cytosol Cyt-c protein expression in acute lung injury rats (**  **±s, n = 8)**

Group	Cyt-c (mitochondria) (Grayscale scan value)	Cyt-c (cytosol) (Grayscale scan value)	β-actin (Grayscale scan value)
**control group**	178.51	96.992	171.47
**LPS injury group**	41.177**	293.45**	183.66
**L**	62.846^**#**^	226.91^**#**^	182.52
**M**	88.344^**##**^	203.38^**##**^	196.02
**H**	115.27^**##**^	111.84^**##**^	169.28

**P < 0.01,compared to the control group; ^#^ *P* < 0.05, ^##^ *P* < 0.01, compared to the LPS injury group; L:LPS + low-dose NaHS group; M:LPS + middle-dose NaHS group;H: LPS + high-dose NaHS group.

The lung mitochondrial Cyt-c protein expression was significantly decreased in the LPS injury group compared to the control group (*P* < 0.01). In three LPS + NaHS groups, lung mitochondrial Cyt-c protein expression was markedly increased compared to the LPS injury group (*P* < 0.05 or *P* < 0.01). The cytosol Cyt-c protein expression was significantly increased in the LPS injury group compared to the control group (*P* < 0.01). In three LPS + NaHS groups, cytosol Cyt-c protein expression was markedly decreased compared to the LPS injury group (*P* < 0.05 or *P* < 0.01).

## Discussion

ALI/ARDS is a common clinical illness. The current ALI mortality rate is as high as 35%-40% and reaches to 50% in ARDS [26, 27]. ALI’s pathogenesis is complex, and there is still much controversy to be had before we reach a definitive conclusion [28]. Increased oxidative stress has been implicated in its pathogenesis [29, 30]. The lung-protective, low-tidal-volume ventilation strategy increases survival rate by limiting alveolar damage and consequent biotrauma [31], but ideal targeted drug therapies have been shown to be curative. Therefore, alternative strategies are urgently needed to improve care. The LPS-induced ALI rat model is a classic animal model [5]. In our ALI rat model, LPS-treated rats showed higher IQA scores than control rats, which is in compliance with ALI’s clinical manifestations [32]. Thus, injecting LPS via the sublingual vein successfully induced ALI in rats, as reported previously [5].

In recent years, some researchers found that mitochondrial dysfunction plays an important role in the course of ALI [33]. Mitochondria utilize approximately 98% of total body oxygen consumption [34, 35]. This would maintain tissue oxygen levels by decreasing demand and protect against cell death [36]. Studies have shown that mitochondrial dysfunction is the key factor to cell damage [37]. In sepsis, mitochondrial dysfunction in vital organs can make cell organisms lack energy, causing multiple organ failure [38]. The lung is a special organ relatively susceptible to injury [39]. The mitochondrion is a complex and sensitive organelle. ALI can lead to abnormal mitochondrial structure and function and tends to cause abnormal mitochondria organelles or other changes in the entire cell, thereby increasing the degree of ALI. Meanwhile, mitochondrial dysfunction is also prone to result in ALI/ARDS [40].

Including the case of LPS-induced lung injury, lung tissue can produce large radical NO, O^-2^, and ONOO^-^. The mitochondrial film, which is rich in unsaturated fatty acids, is a major free radical attack target. These lead to mitochondrial membrane swelling expansion, lipid oxidation increase, and decrease membrane fluidity. Mitochondrial ATP enzyme activity and mitochondrial ATP production were also decreased because of these. Mitochondria have intrinsic defense mechanisms to protect against ROS-induced damage through its large array of antioxidants (e.g., superoxide dismutase, glutathione, thioredoxin) [41]. Under physiological conditions, mitochondrial SOD and GSH-Px content are rich. They timely remove oxygen free radicals. In ALI, cells produce large amounts of oxygen free radicals, resulting in mitochondrial SOD and GSH-Px activities. ATP production decreases while consumption and MDA content increase. Ultimately, these lead to mitochondrial structure destruction [42].

H_2_S is a gaseous mediator that may be a promising therapy for preserving organ function and life during suspended animation *in vivo* models [43]. Major targets clearly include the inhibition of mitochondrial cytochrome c oxidase and activation of endothelial cell K^+^-ATPase channels, but the downstream effects of both pathways are highly context-dependent. Potential utility of H_2_S in sepsis has been demonstrated in several animal studies with improvements in organ function and survival [44–46]. Our experimental results showed that, compared to the control group, IQA scores, W/D weight ratios, IL-1β serum content, lung mitochondrial MDA content, and mitochondrial swelling in the LPS injury group were significantly increased, and mitochondria, ATPase, SOD, and GSH-Px activities were significantly decreased. The lung mitochondrial Cyt-c protein expression was significantly decreased, the cytosol Cyt-c protein expression was significantly increased (Cyt-c was released from mitochondria), and the lung mitochondrial ultrastructure was damaged. Compared to the LPS injury group, the NaHS treatment group’s IQA scores, W/D weight ratio, IL-1β serum content, mitochondrial MDA content, and mitochondrial swelling were significantly decreased, and mitochondria, ATPase, SOD, and GSH-Px activities were significantly increased. The lung mitochondrial Cyt-c protein expression was significantly increased, the cytosol Cyt-c protein expression was significantly decreased (suppressed the release of Cyt-c from mitochondria), and the lung mitochondrial ultrastructure damage looked better. Meanwhile, these therapeutic roles were dose-dependent (positively correlated with the dosage). The mechanism for this could be that H_2_S suppressed the mitochondrial cytochrome c oxidase, decreased the level of mitochondrial lipid peroxidation, and protected cell structure. However, conflicting data exists in models of cecal ligation and puncture demonstrating aggravation [47, 48]. It is likely that H_2_S donor purity, administration route, timing, and dosage may be accountable for the inconsistent data. Our experiment has confirmed that H_2_S can reduce mitochondrial oxidative damage by free radicals, thereby reducing lung tissue damage in ALI and playing a therapeutic role in a dose-dependent manner.

### Limitations

The most common experimental ALI models are caused by sepsis, including injection (local or systemic) of bacterial products like endotoxin (LPS), a commonly used organism called Escherichia coli, or lipoteichoic acid. Other models include extrusion of fecal contents from surgically manipulated gut areas [usually cecum, cecal ligation, and puncture (CLP)]. The colon ascendens stent peritonitis (CASP) causes an intraperitoneal leak of feces, leading to polymicrobial sepsis, similar to what is seen in the CLP model. Each of these models has advantages and disadvantages with dramatic differences between response timing (such as when cytokine/chemokine levels peak in plasma). In rodents injected with LPS, plasma mediators peak in the first several hours versus in CLP, with mediator peaks developing much more slowly over the first 48 h. In future studies, we will also use other models.

Lung tissue H_2_S concentrations were not measured in this study. Comparing the lung tissue concentrations of sulfide species in future studies may help explain the differential biological effects of intravascular NaHS and its dose-dependency.

A recent study [49] suggested that H_2_S may protect the lung against injury and drive epithelial cell migration and wound repair. This idea is supported by the increase of the activated Akt fraction (Protein kinase B). Our study does not involve the impact of H_2_S on Akt pathway activation.

## Conclusions

In our model, LPS resulted in lung mitochondrial structure injury and loss of function in ALI rats. Hydrogen sulfide decreased lung water, decreased the level of inflammation, reduced LPS-induced lung mitochondrial oxidative damage, and protected mitochondrial structure and function. This therapeutic role is dose-dependent. Its regulatory effect on lung mitochondria is positively correlated with the dose. Further studies are needed to reveal its potential mechanism.

### Key messages

- The *in vivo* experimental study demonstrated that hydrogen sulfide (considered as the third gaseous transmitter) donor, NaHS, can prevent mitochondrial oxidative damage and protect mitochondrial structure and function in LPS-induced ALI rat models.
- NaHS reduced ALI-induced oxidative stress.
- NaHS reduced ALI-induced mitochondrial dysfunction.
- NaHS up-regulated the lung mitochondrial Cyt-c protein expression and down-regulated the cytosol Cyt-c protein expression (suppressed the release of Cyt-c from mitochondria) in this model.
- NaHS reduced ALI-induced mitochondrial structural damage.
- Regulatory effect of NaHS on lung mitochondria is positively correlated with the dose.

## Abbreviations

ALI: Acute lung injury
ARDS: Acute respiratory distress syndrome
LPS: Lipopolysaccharide
SIRS: Systemic inflammatory response syndrome
PMN: Polymophonuclear neutrophil
NO: Nitric oxide
CO: Carbon monoxide
H_2_S: Hydrogen sulfide
NaHS: Sodium hydrosulfide
ROS: Radical oxygen species
OONOO-: Peroxynitrite
IQA: Index of quantitative assessment
MDA: Malondialdehyde
SOD: Superoxide dismutase
ATPase: Adenosine triphosphatease
GPx: Glutathione peroxidase
Cyt-c: Cytochrome c
Akt: Protein kinase B
SD: Standard deviation.
